# Stokesian dynamics simulations of a magnetotactic bacterium

**DOI:** 10.1140/epje/s10189-021-00038-5

**Published:** 2021-03-23

**Authors:** Sarah Mohammadinejad, Damien Faivre, Stefan Klumpp

**Affiliations:** 1 Institute for the Dynamics of Complex Systems, University of Göttingen, Friedrich-Hund-Platz 1, 37077 Göttingen, Germany; 2 Department Theory and Bio-Systems, Max Planck Institute of Colloids and Interfaces, 14424 Potsdam, Germany; 3 Department of Biological Sciences, Institute for Advanced Studies in Basic Sciences (IASBS), Zanjan, 45137-66731 Iran; 4 Department of Biomaterials, Max Planck Institute of Colloids and Interfaces, 14424 Potsdam, Germany; 5 Aix-Marseille Université, CEA, CNRS, BIAM, 13108 Saint-Paul-lez-Durance, France

## Abstract

**Abstract:**

The swimming of bacteria provides insight into propulsion and steering under the conditions of low-Reynolds number hydrodynamics. Here we address the magnetically steered swimming of magnetotactic bacteria. We use Stokesian dynamics simulations to study the swimming of single-flagellated magnetotactic bacteria (MTB) in an external magnetic field. Our model MTB consists of a spherical cell body equipped with a magnetic dipole moment and a helical flagellum rotated by a rotary motor. The elasticity of the flagellum as well as magnetic and hydrodynamic interactions is taken into account in this model. We characterized how the swimming velocity is dependent on parameters of the model. We then studied the U-turn motion after a field reversal and found two regimes for weak and strong fields and, correspondingly, two characteristic time scales. In the two regimes, the U-turn time is dominated by the turning of the cell body and its magnetic moment or the turning of the flagellum, respectively. In the regime for weak fields, where turning is dominated by the magnetic relaxation, the U-turn time is approximately in agreement with a theoretical model based on torque balance. In the strong-field regime, strong deformations of the flagellum are observed. We further simulated the swimming of a bacterium with a magnetic moment that is inclined relative to the flagellar axis. This scenario leads to intriguing double helical trajectories that we characterize as functions of the magnetic moment inclination and the magnetic field. For small inclination angles ($\lesssim {20^{\circ }}$) and typical field strengths, the inclination of the magnetic moment has only a minor effect on the swimming of MTB in an external magnetic field. Large inclination angles result in a strong reduction in the velocity in direction of the magnetic field, consistent with recent observations that bacteria with large inclination angles use a different propulsion mechanism.

**Graphic abstract:**

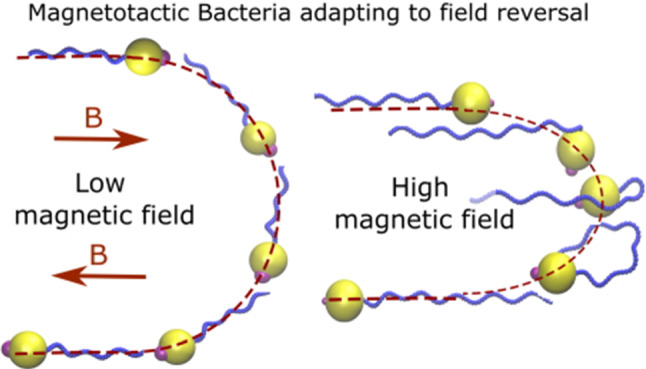

**Supplementary Information:**

The online version supplementary material available at 10.1140/epje/s10189-021-00038-5.

## Introduction

The swimming of motile microorganisms is of great scientific interest, for understanding motility in natural systems, as instances of active matter with all its intriguing physical properties and as inspiration for the development of synthetic microswimmers and microrobots [1–3]. Microorganisms use diverse propulsion mechanisms and behavioral strategies in their swimming. While some of them such as the run-and-tumble motion of *Escherichia coli* [4, 5] or the swimming sperm [6] (and in both cases, their use in chemosensing) are well studied and understood in great detail, other mechanisms of swimming and the corresponding strategies for sensing and steering have only been described recently [7–9]. Among microorganisms, magnetotactic bacteria (MTB) are of particular interest because they can be steered by a magnetic field [10, 11], which provides a promising biocompatible option for the remote control of swimming. MTB are a diverse group of microorganisms with the ability to orient and migrate along (geo-)magnetic field lines [10, 12, 13]. This ability is based on the presence of an intracellular structure called magnetosome chain, which acts as a magnetic compass for their navigation. The chain is composed of iron oxide or iron sulfide nanoparticles enclosed in membranous vesicles, called magnetosomes, which imparts a magnetic moment to MTB [14]. Following the Earth’s magnetic field lines is hypothesized to help them find their preferred habitat near the oxic-anoxic transition zone in stratified aquatic environment and sediments [10]. This type of aerotaxis, which is assisted by magnetic field is called magnetoaerotaxis . From a physical point of view, magnetotaxis can either provide a direction or only an axis, but in either case reduces a three-dimensional search process to a one-dimensional search along the direction defined by the field [15, 16].

In recent years, MTB have attracted the attention of researchers from many different disciplines [11], including from an application point of view. MTB have been functionalized for their use as a magnetically targeted drug delivery system [17]. They also provide inspiration for synthetic and biohybrid magnetic transport: for example, magnetic guidance has been demonstrated after incorporation or attachment of magnetic particles to naturally non-magnetic organisms [18, 19] and synthetic magnetically steered swimmers were constructed with the purpose of finding the best structure for rapid swimming while being steering by magnetic field [20–22].

Despite the promising proof-of-concept applications of MTB, many aspects of the dynamics of MTB are only beginning to be known such as details of their swimming mechanisms, their strategies for responding to environmental stimuli and changing the direction of motion. Since MTB are rather diverse with respect to cell shapes, magnetic dipole orientation and number and organization of their flagella, a variety of mechanisms and strategies are expected to be observed [15].

Few experimental studies have addressed the detailed mechanisms of propulsion of MTB by their flagella. Notable exceptions are studies on the coordination of two flagella at opposite poles in *Magnetospirillum magneticum* (AMB-1) [23], of two velocity modes in *Magnetospirillum gryphiswaldense* (MSR-1) [24] and of the cooperation of two flagellar bundles almost perpendicular to the magnetic moment in *Magnetococcus marinus* (MC-1) [9]. These studies are complemented by theoretical approaches. Here simple models describe the bacteria either as self-propelled dipolar particles [25–27] or as a cell body with a rigid rotating helix [28–30] and did not include the hydrodynamics and/or mechanics of the flagellum. In this paper, we use Stokesian dynamics, which provides an efficient framework to carry out numerical simulations for particles suspended in a fluid that interact through hydrodynamic and other forces [31]. Stokesian dynamics has been used extensively for the study of the swimming of non-magnetotactic bacteria, in particular for the swimming of *Escherichia coli* including different dynamical states of its flagellum and the hydrodnyamic interaction between its multiple flagella [32–34]. The same model was used to investigate the origin of screw formation in the flagellum in single-flagellated *Shewanella putrefaciens* attempting to escape from the traps [8]. Likewise, in our recent work on the *Magnetococcus marinus*, we used the same approach as a complement to experimental three-dimensional tracking of swimming bacteria to distinguish different swimming modes and to propose that the two flagella of this bacterium rotate in opposite direction [9].

Here, we report on simulations of the swimming behavior of a model MTB with spherical cell body and a single flagellum. We characterized the influence of various parameters such as the flagellum length, the orientation of the magnetosome chain relative to the flagellar axis and the strength of the magnetic field. Specifically, we present two scenarios that are relevant from an experimental point of view: we investigated the U-turns that are observed when the magnetic field is reversed and asked whether an inclination of the magnetic moment relative to the flagellar propulsion axis is detrimental to the swimming motion.

## Method

**Fig. 1:**
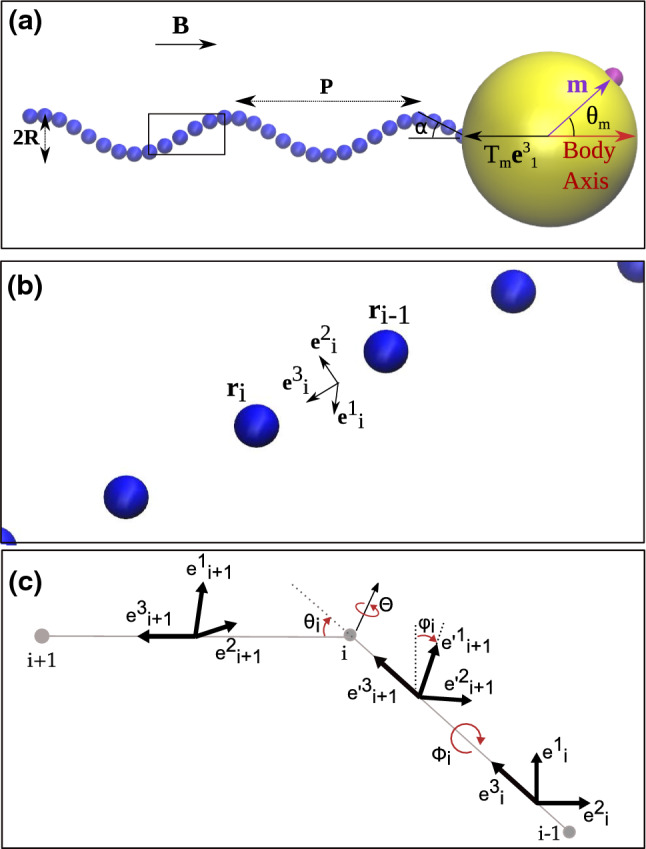
**a** Model of a magnetotactic bacterium with one flagellum. The flagellum and the cell body are shown in blue and yellow, respectively. The magnetic moment and the motor rotation axis are shown with purple and black arrows on the cell body. The flagellum is modeled as a discretized space curve composed of *N* particles. A part of the flagellum is magnified in **b** to indicate the triad vectors defined between two successive particles of the flagellum. **c** Representation of the bending and twist angles ($\theta _i$ and $\phi _i$) and the corresponding deformation of the triads along the flagellum length. The primed triad represents the intermediate state after the twist is applied, but before bending

In this paper, we used Stokesian dynamics simulations to study the swimming behavior of a single-flagellated magnetotactic bacterium. We present a semi-realistic model for MTB taking into account its magnetic orientation and its propulsion by the flagellar rotation. In the model, the cell body is spherical and has a single flagellum discretized as a chain of beads (shown in Fig. 1a, with yellow and blue colors, respectively). The flagellum, in turn, consists in a helical filament, a rotary motor embedded in the cell membrane, which rotates the filament, and a short hinge for transmitting the motor torque to the filament [33]. The description of the flagellum includes its elasticity, as well as hydrodynamic and excluded volume interactions. The hydrodynamic interactions are taken into account by calculating the mutual mobility coefficients between particles representing segments of the flagellum and then solving the equations of motion for all particles using these mobility coefficients [35].

### Model of the flagellum

The model we used for the flagellum of our MTB is the model that has been used for the *E. coli* flagellum by Vogel et al. [20, 32]. In this model, the helical filament of the flagellum is described as a discretized space curve composed of *N* particles of diameter $a = 0.02\,\upmu \hbox {m}$ and equilibrium separation distance $L_e = 0.2\,\upmu \hbox {m}$ (Fig. 1a). In order to explore the bacterial behavior as a function of the flagellum length, *N* was varied between 20 and 150. As shown in Fig. 1b, the position of each flagellum particle is represented by ${\mathbf {r}}_i$ $(i=1, .. , N)$. A set of three orthogonal unit vectors ${\mathbf {e}}^{\alpha }_i = \lbrace {\mathbf {e}}^1_i, {\mathbf {e}}^2_i, {\mathbf {e}}^3_i\rbrace$ (called the *i*-th triad with $\alpha = 1, 2, 3$ representing the three orthogonal unit vectors) is assigned to each particle *i* to describe the orientation and elastic deformation of the bond between two successive particles *i* and $i-1$ (Fig. 1b). The first triad ${\mathbf {e}}^{\alpha }_1$ is associated with the bond between the cell body and the first flagellar particle.

A regular helical filament like the one we have for the flagellum can be characterized completely by its constant curvature ($\kappa _e$) and torsion ($\tau _e$) which are related to the helix radius (*R*) and pitch (*P*) via $R = \frac{\kappa _e}{\kappa _e^2+\tau _e^2}$ and $P = \frac{2\pi \tau _e}{\kappa _e^2+\tau _e^2}$ [36].

Since the flagellum is an elastic filament, any deviation from its equilibrium configuration costs energy. The elastic energy for the bending and twisting of the flagellum ($H^{K}$) can be calculated by regarding the conformational deviation from the helical equilibrium configuration using a discretized version of Kirchhoff’s theory for elastic rods [37],

1 $$\begin{aligned} H^{K}&= L_e\displaystyle \sum _{i=1}^{N-1} \lbrace A[(\varOmega ^1_i-\varOmega ^1_e)^2+(\varOmega ^2_i-\varOmega ^2_e)^2] \nonumber \\&\quad + C(\varOmega ^3_i-\varOmega ^3_e)^2\rbrace , \end{aligned}$$

in which $\varOmega ^\alpha _i = \lbrace \varOmega ^1_i, \varOmega ^2_i, \varOmega ^3_i\rbrace$ is the instantaneous local angular strain vector. Its components are related to the local curvature and torsion angles and the local triads via $\varOmega ^1_i = -\frac{\theta _i}{\sin \theta _i}{\mathbf {e}}^2_i.{\mathbf {e}}^3_{i+1}$, $\varOmega ^2_i = \frac{\theta _i}{\sin \theta _i}{\mathbf {e}}^1_i.{\mathbf {e}}^3_{i+1}$, $\varOmega ^3_i = \phi _i$, where $\theta _i$ and $\phi _i$ (as shown in Fig. 1c) are the bending and twist angles between successive triads, respectively. *A* and *C* are the bending and torsional rigidities, for which the values of $A = C = 3.5\,{\hbox {pN}\,\upmu \hbox {m}^2}$ are used.

$\varOmega ^\alpha _e = \lbrace \varOmega ^1_e, \varOmega ^2_e, \varOmega ^3_e\rbrace = \lbrace \kappa _e \cos (L_e\tau _e), \kappa _e \sin (L_e\tau _e), \tau _e\rbrace$ is the equilibrium angular vector for the flagellum. Throughout this paper, we use $\kappa _e = 1.3\,{\upmu \hbox {m}^{-1}}$, $\tau _e = -2.1\,{\upmu \hbox {m}^{-1}}$, except in the section where we study the effect of the flagellum helical geometry on the swimming velocity (Fig. 3b) where we run simulation for different $\varOmega ^\alpha _e$.

The corresponding elastic forces on *i*-th particle (${\mathbf {F}}^{K}_i$) and torques about *i*-th triad (${\mathbf {T}}^{K}_i$) can be obtained by calculating the numerical derivatives of $H^{K}$ with respect to the position of the particle (${\mathbf {r}}_i$) and the twist angle of the triads ($\phi _i$) as follows,

2 $$\begin{aligned} {\mathbf {F}}^{K}_i = -\frac{\partial H^{K}}{\partial {\mathbf {r}}_i}, \end{aligned}$$

3 $$\begin{aligned} T^{K}_i = -\frac{\partial H^{K}}{\partial \phi _i}. \end{aligned}$$

The stretching elasticity of the flagellum is taken into account using a Hookian free energy ($H^{S}$) of the form

4 $$\begin{aligned} H^{S} = \frac{K_s}{2L_e}\displaystyle \sum _{i=2}^N (L_i-L_e)^2, \qquad L_i = \vert {\mathbf {r}}_i - {\mathbf {r}}_{i-1} \vert \end{aligned}$$

with stretching rigidity of $K_s = 1000{\hbox {pN}}$ [33]. The stretching force (${\mathbf {F}}^{S}_i$) can be obtained by analytical differentiation of $H^{S}$ with respect to ${\mathbf {r}}_i$. The resulting ${\mathbf {F}}^{S}_i$ is

5 $$\begin{aligned} {\mathbf {F}}^{S}_i = -\frac{K_s}{L_e} \lbrace (L_i-L_e)\mathbf{e }^3_i - (L_{i+1}-L_e)\mathbf{e }^3_{i+1}\rbrace . \end{aligned}$$

The repulsive part of a Lennard-Jones force (a WCA force) is used to model excluded volume interactions between all particles including the cell body ($i=0$),

6 $$\begin{aligned} \mathbf{F }^{LJ}_{i} = \left\{ \begin{array}{l l} \frac{F_0}{\sigma }\ \left[ 2\left( \frac{\sigma }{r_{ij}}\right) ^{14}-\left( \frac{\sigma }{r_{ij}}\right) ^{8}\right] \mathbf{r }_{ij} &{} \hbox { if}\ r_{ij}<\root 6 \of {2}\sigma ,\\ 0 &{} \hbox { if}\ r_{ij} \ge \root 6 \of {2}\sigma , \end{array} \right. \nonumber \\ \end{aligned}$$

with $F_0 = 1\hbox {pN}$. We set $\sigma =2a$ for the interaction between two (non-bonded) beads of the flagellum, so that the distance between two beads on which the steric interactions become relevant is 2*a*. For the interaction between flagellum beads and the cell body, we use $\sigma = a+R_b$. $\mathbf{r }_{ij}$ is the distance vector between *i*-th and *j*-th particles (including the cell body ($i=0$)) and $r_{ij}$ its absolute value. The steric force on the *i*-th particle (${\mathbf {F}}^{LJ}_i$) is calculated by summation of the steric forces from all other particles (*j*) on the *i*-th particle. We note that despite the relatively large gaps between subsequent flagellar beads ($L_e = 10a$), we do not observe beads passing through the flagellum due to its rather high bending and twist rigidities.

The filament is attached to the surface of the bacterial cell body via its first particle ($i=1$). We defined the first triad (${\mathbf {e}}^{\alpha }_1$) in such a way that its third component (${\mathbf {e}}^3_1$) is along the vector drawn from body center to the first flagellar particle attached to body ($i=1$-th particle). The effect of the rotary motor was modeled by the motor torque $T_m {\mathbf {e}}^3_1$, for which the value $T_m = 3.4\,{\hbox {pN}\,\upmu \hbox {m}}$ was assumed, acting on the first ($i=1$) bead of the flagellum. This torque is transmitted to the main part of the filament via the hinge, represented by the connection to the second bead. For the latter we used the rigidities $A^{\prime } = A$ and $C^{\prime } = 3C$. The high torsional rigidity provides the coupling of the filament to the motor rotation.

### Dynamics of the flagellum

At low Reynolds numbers, because of the linearity of Stokes’ equation, the translational and angular velocity vectors (${\mathbf {v}}$ and $\pmb {\omega }$, respectively) of the particles have a linear relation with the forces (${\mathbf {F}}$) and torques (${\mathbf {T}}$) acting on those particles as follows

7 $$\begin{aligned} \begin{bmatrix} \mathbf{v }\\ \pmb {\omega } \end{bmatrix} = \begin{bmatrix} {\pmb {\mu }}^{tt} &{} {\pmb {\mu }}^{tr}\\ {\pmb {\mu }}^{rt} &{} {\pmb {\mu }}^{rr} \end{bmatrix} \begin{bmatrix} \mathbf{F }\\ \mathbf{T } \end{bmatrix}. \end{aligned}$$

Here $\mathbf{v }$ and $\pmb {\omega }$ are 3*N*-component linear and angular velocity vectors. The matrix is called the mobility matrix and $\pmb {\mu }^{tt}$, $\pmb {\mu }^{rr}$, $\pmb {\mu }^{rt}$ and $\pmb {\mu }^{tr}$ are each $3N \times 3N$ mobility sub-matrices representing the translational–translational, rotational-rotational and rotational–translational coupling between velocities and forces of all particles. In the following we refer to the mobility terms between force and velocity of the same particle as self-mobilities and those between forces and velocities of different particles as cross-mobilities.

In this study, the rotational–translational mobility sub-matrices ($\pmb {\mu }^{tr}$ and $\pmb {\mu }^{rt}$) and rotational-rotational cross-mobility terms ($\pmb {\mu }^{rr}_{i\ne j}$) were neglected. This is mostly done for technical reasons, since these couplings may result in the rotation of the triads and cause inconsistencies with the definition of the bond direction ${\mathbf {e}}^3_i = r_i-r_{i-1}$. Artifacts were reported in earlier work, specially for elastic structure [38]. From the physical point of view, these contributions can be neglected because the $\pmb {\mu }^{rr}_{ij}$ and $\pmb {\mu }^{tr}_{i\ne j}$ terms in the Rotne–Prager expansion are of higher order in $\frac{a}{r_{ij}}$ compared to $\pmb {\mu }^{rr}_{ii}$. The remaining mobility terms for flagellar particles are the translational–translational cross-mobility ($\pmb {\mu }^{tt}_{i\ne j}$), the translational–translational self-mobility ($\pmb {\mu }^{tt}_{ii}$) and the rotational-rotational self-mobility ($\pmb {\mu }^{rr}_{ii}$). The translational–translational cross-mobilities ($\pmb {\mu }^{tt}_{i\ne j}$) between flagellar particles were calculated using the Rotne–Prager approximation [35, 39],

8 $$\begin{aligned} \pmb {\mu }^{tt}_{i\ne j} = \mu ^t \begin{bmatrix} \frac{3}{4}\frac{a}{r_{ij}} (\mathbf{1 }+\hat{\mathbf{r }}_{ij}\otimes \hat{\mathbf{r }}_{ij})+\frac{1}{2} (\frac{a}{r_{ij}})^3(\mathbf{1 }-3\hat{\mathbf{r }}_{ij}\otimes \hat{\mathbf{r }}_{ij}) \end{bmatrix}.\nonumber \\ \end{aligned}$$

Here $r_{ij}$ represents again the distance between the *i*-th and *j*-th particles. For the translational–translational self-mobility ($\pmb {\mu }^{tt}_{ii}$, a $3\times 3$-submatrix of $\pmb {\mu }^{tt}$) of the flagellar particles we used the mobility relation for a straight rod that is

9 $$\begin{aligned} \pmb {\mu }^{tt}_{ii} = {\mathbf {e}}^3_i\otimes {\mathbf {e}}^3_i / \gamma _{\parallel } +({\mathbf {1}} - {\mathbf {e}}^3_i\otimes {\mathbf {e}}^3_i) / \gamma _{\perp }, \end{aligned}$$

in which $\gamma _{\parallel } = 3.2 \times 10^{-4}\,{\hbox {pNs}/\upmu \hbox {m}}$ and $\gamma _{\perp } = 5.6 \times 10^{-4}\,{\hbox {pNs}/\upmu \hbox {m}}$ were used as the anisotropic friction coefficients of the flagellum following the values used for the *E. coli* flagellum [33]. The rotational-rotational self-mobility of flagellar particles ($\mu ^{rr}_{ii}$) is the inverse of the rotational friction $\mu ^{rr}_{ii} = 1/\gamma _r$ with $\gamma _r = 2.52 \times 10^{-7}\,{\hbox {pNs}\,\upmu \hbox {m}}$.

Taking into account the forces and torques discussed above and including them in Eq. (7), the dynamics of the flagellar particles’ positions (${\mathbf {r}}_i$) and triads (${\mathbf {e}}^{\alpha }_{i}$ with $\alpha = 1, 2, 3$ can be obtained by solving the following equations of motion for their position and orientation,

10 $$\begin{aligned} \frac{\partial {\mathbf {r}}_i}{\partial t}= & {} \mathbf {\mu }^{tt}_{ij}({\mathbf {F}}^{K}_j + {\mathbf {F}}^{S}_j + {\mathbf {F}}^{LJ}_j),   i = 2,..,N\nonumber \\ \end{aligned}$$

11 $$\begin{aligned} \frac{\partial {\mathbf {e}}^{\alpha }_{i}}{\partial t}= & {} \mu ^{rr}_{ii} (T^{K}_i{\mathbf {e}}^{3}_i\times {\mathbf {e}}^{\alpha }_i),   i = 2,..,N\nonumber \\ \end{aligned}$$

12 $$\begin{aligned} \frac{\partial \mathbf {e_f}^{\alpha }_{1}}{\partial t}= & {} \mu ^{rr}_{11} (T^{K}_1+T_m ) ({\mathbf {e}}^{3}_1\times {\mathbf {e}}^{\alpha }_1), \end{aligned}$$

in which $\frac{\partial \mathbf {e_f}^{\alpha }_{1}}{\partial t}$ is the contribution of the flagellum torques in the dynamics of the first triad. Another contribution $\frac{\partial \mathbf {e_b}^{\alpha }_{1}}{\partial t}$ comes from the torques on the cell body (Sect. 2.3 Eq. (15)). These two terms add up to the total change of ${\mathbf {e}}^{\alpha }_{1}$ as follows

13 $$\begin{aligned} \frac{\partial {\mathbf {e}}^{\alpha }_{1}}{\partial t}=\frac{\partial \mathbf {e_f}^{\alpha }_{1}}{\partial t}+\frac{\partial \mathbf {e_b}^{\alpha }_{1}}{\partial t}. \end{aligned}$$

### Dynamics of the cell body

To obtain the dynamics of the whole bacterium, the equations of motion for the cell body are also needed and have to be solved simultaneously. For simplicity, we modeled the cell body with a sphere of radius $R_b = 1\,\upmu \hbox {m}$. Since MTB contain a magnetic chain, which acts as a compass needle, we defined a magnetic moment vector (${\mathbf {m}}$) for the cell body (shown in Fig. 1a) with a magnitude of $m = 6.2 \times 10^{-16}{\hbox {Am}^2}$ [40]. In each simulation the direction of ${\mathbf {m}}$ with respect to the body axis is fixed and its inclination is represented by an angle $\theta _m$. We should note that ${\mathbf {m}}$ is oriented along the body axis ($\theta _m = {0^{\circ }}$) except for the section where we study the effect of the inclination of ${\mathbf {m}}$ with respect to body axis ($\theta _m \ne 0^{\circ }$) explicitly. In an external magnetic field ${\mathbf {B}}$, the equations of motion for the cell body are

14 $$\begin{aligned}&\frac{\partial \mathbf {r_b}}{\partial t} = \mu ^{tt}_b \left[ {\mathbf {F}}^{K}_1 + {\mathbf {F}}^{S}_1+ {\mathbf {F}}^{LJ}_0\right] , \end{aligned}$$

15 $$\begin{aligned}&\frac{\partial \mathbf {e_b}^{\alpha }_1}{\partial t}= \mu ^{rr}_b \left[ {\mathbf {m}}\times {\mathbf {B}} + (\mathbf {r_1}-\mathbf {r_b})\right. \nonumber \\&\qquad \quad \times \left. ({\mathbf {F}}^{K}_1+{\mathbf {F}}^{S}_1) \right] \times {\mathbf {e}}^{\alpha }_1, \end{aligned}$$

16 $$\begin{aligned}&\frac{\partial {\mathbf {m}}}{\partial t} = \mu ^{rr}_b \left[ {\mathbf {m}}\times {\mathbf {B}} + (\mathbf {r_1}-\mathbf {r_b})\right. \nonumber \\&\quad \times \left. ({\mathbf {F}}^{K}_1+{\mathbf {F}}^{S}_1) - T_m {\mathbf {e}}^3_1\right] \times {\mathbf {m}}. \end{aligned}$$

Here $\mu ^{tt}_b = \frac{1}{6 \pi \eta R_b}$ and $\mu ^{rr}_b= \frac{1}{8\pi \eta R^3_b}$ are the translational–translational and rotational-rotational mobility coefficients of the cell body in which $\eta$ is the viscosity of water. Cross-mobility terms between the cell body and the flagellar particles were neglected.

Equation (14) describes the translational motion of the cell body. The force on the first flagellar particle (${\mathbf {F}}^{K}_1 + {\mathbf {F}}^{S}_1$) is transmitted directly to the cell body, together with the Lennard-Jones force (${\mathbf {F}}^{LJ}_0$). Eq. (15) describes the contribution of the cell body rotation on the evolution of ${\mathbf {e}}^{\alpha }_1$, the triad associated with the bond between the cell body and the first flagellar particle. Its terms are the magnetic torque and the torque resulting from the forces acting on the first bead, respectively. As mentioned earlier, Eq. (12) and (15) add up to the total change of the first triad as given by Eq. (13).

The magnetosome chain has a fixed orientation in the cell body, so the magnetic moment ${\mathbf {m}}$ rotates rigidly with the cell body. To determine the orientation of the cell body, we need to include one additional equation of motion in the model that describes the dynamics of an arbitrary vector fixed on the cell body. We used the equation for the magnetic moment as the equation for finding the orientation of the cell body at each time-step. Eq. (16) represents the time evolution of ${\mathbf {m}}$ and the torque terms in its right-hand side are the magnetic toque, the torque arising from the force on the first particle and the counter torque to motor rotation ($-T_m {\mathbf {e}}^3_1$).

Since the first flagellum bead $(i = 1)$ is rigidly attached to the cell body, its position at each time-step is determined by $\mathbf {r_1} = \mathbf {r_b}+R_b {\mathbf {e}}_1^3$.

### Implementation of the model

The model was implemented using a self-written C++ program. The equations of motion (Eqs. (10) to (16)) were solved numerically using second-order Runge–Kutta algorithm [41, 42], specifically Heun method [43], to obtain the successive configurations of beads position and triads at each time-step knowing their previous configuration. For simplicity, we chose the magnetic field to be in *z*-direction and its magnitude in our simulation was varied from zero to hundred times the Earth magnetic field ($B_E = 50\,{\upmu \hbox {T}}$).

We used a time-step of $\varDelta t = 4\times 10^{-9}{\hbox {s}}$ for all simulations and an initial configuration (helical shape) of the flagellum close to its equilibrium state. Nevertheless, equilibration takes of the order of $\times 10^7$ time steps. Therefore, the simulations were run for several $\times 10^7$ steps, with the total duration adjusted to the specific parameters. In particular, in the simulations with an inclined magnetic moment (Sect. 3.4), at least one full helical turn of the trajectories was simulated; thus, longer simulations were needed for large inclination angles, where the helix radius is large. Likewise for U-turn simulations (Sect. 3.3), longer runs were needed for low magnetic fields. In the U-turn scenario, the simulation was run to equilibrate for $5\times 10^6$ time steps before the field was reversed.

## Results and discussion

### Swimming and alignment with magnetic field

**Fig. 2:**
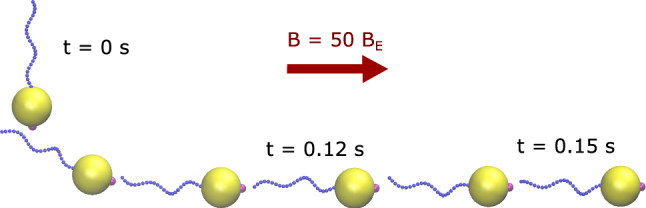
Alignment of the model magnetotactic bacterium in a magnetic field $B = 50 B_E$. The initial configuration of the bacterium is perpendicular to the magnetic field

The Stokesian Dynamics simulation framework allows us to address the mechanisms of swimming while a MTB interacts with a magnetic field. In particular, the model incorporates the magnetic moment of the cell body, which interacts with the field, the helical structure of the flagellum as well as its elasticity and the torque due to the flagellar motor. That the model describes both propulsion and magnetic steering is shown by the following simulation: Our bacterium is put in a uniform magnetic field, aligned initially perpendicular to that field. Running the simulation shows that the model bacterium aligns in the direction of the field by turning while swimming and then continues to swim along that direction. An exemplary reorientation trajectory is depicted in Fig. 2 by illustrating successive snapshots. The corresponding movie is also available in supplementary information (Video 1). Alignment is observed for arbitrary initial orientation and for different field strengths. The time needed for the complete alignment depends on the field strengths. We will discuss the dynamics of alignment in more detail in Sect. 3.3 below.

### Impact of flagellar geometry on swimming

**Fig. 3:**
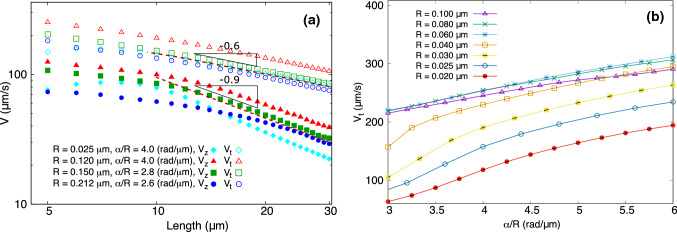
The swimming velocity of the bacterium as a function of geometrical features of flagellum. **a** Double-logarithmic plot of the velocity as a function of the flagellum contour length (*L*) and a linear fit to it, **b** Velocity as a function of the helix parameter $\alpha /R$ for different helix radii (*R*) and pitch angles ($\alpha$) with a constant contour length of $6\,\upmu \hbox {m}$

To characterize our model, we first studied the dependence of the swimming velocity of our model bacterium on geometrical features of the flagellum in the absence of a magnetic field. The geometry of the flagellar helix (as shown in Fig. 1) is defined by its radius *R*, pitch *P*, pitch angle $\alpha$ (with $\tan \alpha = \frac{2\pi R}{P}$) and contour length *L*. We simulated swimming for a wide range of *L* and $\frac{\alpha }{R}$ and determined the average swimming velocity. The swimming velocity as a function of *L* and $\frac{\alpha }{R}$ is plotted in Fig. 3a, b, respectively.

In Fig. 3a, the equilibrium curvature and torsion (and thus *R* and $\alpha$) of the flagellum were kept constant and the length of flagellum (*L*) was modulated by the number of beads *N*. The velocity is plotted as a function of *L* on a double-logarithmic scale. Both the average swimming velocity $(V_z)$ and the tangential velocity $(V_t)$ were calculated in each simulation. Figure 3a shows that the swimming velocity decreases with *L* which is in qualitative agreement with a theoretical prediction [1] for a rigid flagellum,

17 $$\begin{aligned} V\approx \alpha \frac{\xi _{\perp }-\xi _{\parallel }}{\xi _{\parallel }}\frac{\xi _r}{\xi _{\perp }}\frac{R_b^3}{RL}\varOmega _m. \end{aligned}$$

A linear fit to the log–log data (indicated by the dotted lines; the data for short contour lengths were omitted in the fit) shows a decrease as a power law for long flagella, however not with an exponent (slope in the log–log-plot) of $-1$ as predicted by the theoretical expression, but with a slope of $-0.9$. The corresponding slope for the tangential velocity is $-0.6$ (the precise value of the slope depends on the time window of the fit).

This smaller exponent may be attributed to the approximations used in the theory. This formula is derived for the limit of a very long and rigid flagellum and for very small pitch angles $(\alpha \ll 1)$. We also observe in our results that the slope in the log–log plot increases for longer flagella, so it is possibly that it approaches $-1$ for even longer flagella ($>30\,\upmu \hbox {m}$), which are not very realistic as well as computationally expensive. Moreover, for very long flagella, the assumption of a rigid flagellum becomes more and more unrealistic, as the flagellum length exceeds its persistence length. Indeed some simulations for long flagella show very small periodic modulations of the propulsion, likely due to the elasticity of the flagellum. We also note that Eq. (17), which is derived for the limit of long flagella, is not applicable to realistic flagellar lengths ($\sim 10\,\upmu \hbox {m}$).

The simulation results for different sets of the parameters $\lbrace R, \alpha \rbrace$ (Fig. 3a) do not show significant differences in this slope. The velocity as a function of $\frac{\alpha }{R}$ is shown in Fig. 3b for different helix radii. Here the contour length of the flagellum was kept constant at $6\,\upmu \hbox {m}$ and its pitch angle ($\alpha$) was varied. The simulations were carried out for different helix radii in the range of $R = 0.02 {-} 0.12\,\upmu \hbox {m}$. The data show that the velocity increases with increasing $\frac{\alpha }{R}$, as predicted by Eq. (17). The increase is, however, not exactly linear as in that expression. Our simulations indicate that the dominant dependence on the parameters $\alpha$ and *R* is through the ratio $\alpha /R$. Therefore, we varied this parameter in the simulations shown in Fig. 3. However, not all dependence on these two parameters is captured by the $\alpha /R$-dependence, as seen by comparing the data for different *R* with the same ratio $\alpha /R$.

### U-turn in response to magnetic field reversal

If the direction of the magnetic field is rapidly reversed, a swimming MTB performs a U-turn to realign with the magnetic field and swim in the opposite direction. The U-turn trajectory of MTB upon magnetic field reversal is used as a method to measure the magnetic moment of magnetotactic bacteria [44]. The magnetic moment of the bacterium can be calculated from the diameter of the U-turn or the time needed for it. Esquivel et. al [45] have investigated the dynamics of a magnetotactic cell following a sudden reversal of the magnetic field by solving its torque balance equation based on Bean model [46]. Since cells swim at low Reynolds number, inertial terms can be neglected and on average, random forces will create a zero net torque. Therefore, the torque exerted on the dipole by the magnetic field should be balanced by the viscous drag torque, i.e.

18 $$\begin{aligned} m B \sin \theta = \varGamma \frac{\hbox {d}\theta }{\hbox {d}t}, \end{aligned}$$

where *m* is the dipole moment, *B* is the external magnetic filed, $\varGamma$ is the rotational drag coefficient and $\theta$ is the angle between the direction of the magnetic field and the magnetic dipole moment.

This differential equation can be solved for the time it takes for the dipole direction to change from $\theta _i$ to $\theta$ as $\ln (\tan \frac{\theta }{2})=\frac{mB}{\varGamma }t+\ln (\tan \frac{\theta _i}{2})$. Due to thermal fluctuations the initial angle, $\theta _i$, is considered to be very small but not zero (for the onset of field reversal). Therefore, by considering $\theta _f=\frac{\pi }{2}$ (for the middle of U-turn), the U-turn time can be obtained as

19 $$\begin{aligned} \tau _u=-2\frac{\varGamma }{mB}\ln \Big (\tan \frac{\theta _i}{2}\Big ). \end{aligned}$$

Moreover, by integrating the component of velocity along U-turn opening ($\int _0^{\infty } v \sin \theta \hbox {d}t$, where $\sin \theta = \frac{\varGamma }{mB} \frac{\hbox {d}\theta }{\hbox {d}t}$) over time, the U-turn diameter can also be calculated as

20 $$\begin{aligned} D_u=\frac{\pi v \varGamma }{mB}. \end{aligned}$$

**Fig. 4:**
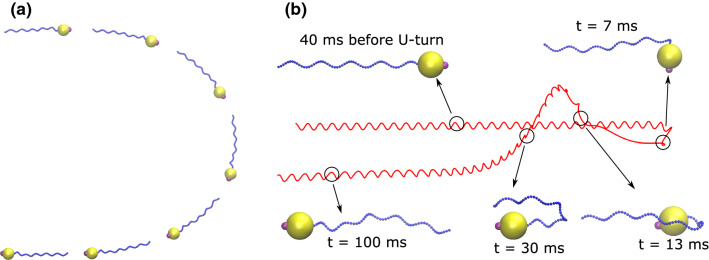
U-turn trajectory illustrated with successive snapshots in case of **a** a weak magnetic field ($10 B_E$) and **b** a strong magnetic field ($500 B_E$) for a bacterium with a flagellum of length $L = 12\,\upmu \hbox {m}$

For our model MTB, we simulated the U-turn experiment for a wide range of magnetic field strength, starting with a bacterium swimming along the direction of the field and instantaneously reversing the field direction. Figure 4a and b shows trajectories and snapshots for two different magnetic field strengths, of $10 B_E$ and $500 B_E$, respectively. Here, the time of the magnetic field reversal is defined as $t = 0{\hbox {s}}$.

With small fields (Fig. 4a), the bacterium aligns with the magnetic field gradually, and thus, an obvious and wide U-turn is observed in its trajectory. However, with large fields (Fig. 4b), the U-turn is very sharp and tight. Moreover, we observe that with large fields, the flagellum undergoes a pronounced bend or kink during the U-turn, as shown in Fig. 4b at $t = 9{\hbox {ms}}$ and $t = 30{\hbox {ms}}$. This kink formation can be attributed to the very fast reorientation of the magnetic moment for alignment with the field. Since the flagellum is long and because of its elasticity, its distant segments can not immediately follow the very fast reorientation of the cell body while the close segments reorient due to their direct attachment to the cell body.

**Fig. 5:**
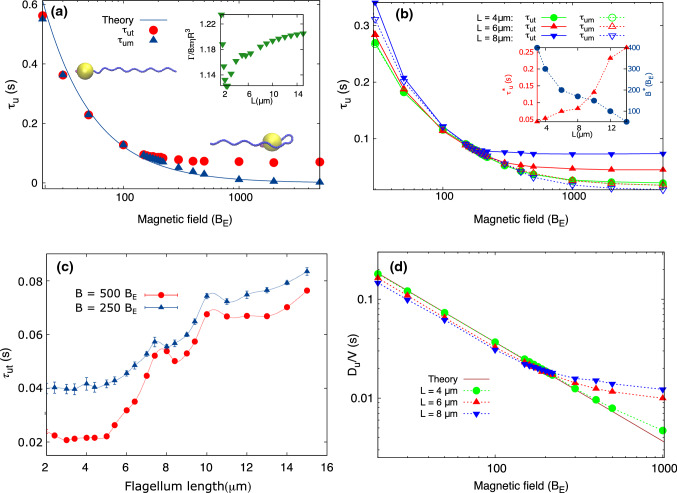
**a** Characteristic time scales of U-turn after field reversal as a function of the magnetic field strength. The times $\tau _{um}$ (blue) and $\tau _{ut}$ (red) measure the time it takes the bacterium to turn its magnetic moments (and thus the cell body) and the time is takes to reverse the swimming direction, respectively. The solid line is a fit to the theoretical expression obtained from torque balance with $\frac{\varGamma }{8\pi \eta R^3}=1.12$ and $\theta _i=0.36$. The flagellum length is $L = 12\, \upmu \hbox {m}$. Inset: The friction coefficient of the whole bacterium as a function of flagellum length obtained from direct simulations in which the bacterium was rotated with an external torque. **b** $\tau _{ut}$ and $\tau _{um}$ as a function of magnetic field for different flagellum lengths of $L = 4, 6, 8\, \upmu \hbox {m}$. Inset: The cross-over field strength ($B^*$) and U-turn time ($\tau _u^*$) between the two regimes dominated by different characteristic times. **c** $\tau _{ut}$ as a function of flagellum length for different strengths of the magnetic field. **d** The U-turn diameter (scaled by swimming velocity) as a function of the magnetic field strength for flagellum lengths of $L = 4, 6, 8\, \upmu \hbox {m}$. The solid line is a fit to the theoretical expression with $\frac{\varGamma }{8\pi \eta R^3}=1.23$

To make this reasoning more quantitative, we determined two characteristic time scales related to the U-turn, the time $\tau _{um}$ after which the magnetic moment is re-aligned with the magnetic field and the time $\tau _{ut}$ after which the swimming direction (and thus the trajectory) is re-aligned with the field direction. The two time scales as obtained from the simulations are plotted as functions of the magnetic field in Fig. 5a.

Figure 5a shows that U-turn time decreases with magnetic field. For fields up to $120 B_E$, both characteristic times have almost the same value, but for stronger fields $\tau _{ut}$ exceeds $\tau _{um}$ remarkably. This confirms our previous interpretation that in these strong fields, the magnetic moment aligns with the field very rapidly, while the flagellum requires more time to align with the cell body’s new direction. In this range of the magnetic field ($B> 120 B_E$), $\tau _{ut}$ plateaus, in agreement with the expectation for a reorientation of the flagellum and thus the trajectory driven by the flagellum’s mechanics rather than by the magnetic torque. Correspondingly, we see that this plateau depends on the length of the flagellum as shown in Fig. 5b. The longer the flagellum, the slower the reorientation (increasing $\tau _{ut}$). $\tau _{um}$, by contrast is not affected by flagellum length. Since the slower of the two processes determines the overall U-turn time, we can define a crossover field strength ($B^*$) between the regimes where magnetic reorientation determines the U-turn time $\tau _u^*$ and a regime where the flagellar reorientation is dominant. This crossover field and the corresponding U-turn time are plotted as functions of flagellum length in the inset of Fig. 5b. Figure 5c shows the U-turn time for strong fields as a function of the flagellum length in more detail. In addition to the overall increase in the $\tau _{ut}$ with the length, we observe an oscillatory pattern with a period that approximately corresponds to the contour length of one turn of the helix ($2.54\,\upmu \hbox {m}$ for the equilibrium curvature and torsion of $\lbrace \kappa _e, \tau _e\rbrace = \lbrace 1.3, -2.1 \rbrace \,{\upmu \hbox {m}^{-1}}$).

The analytical results for the U-turn time and U-turn radius discussed above consider only the magnetic reorientation and are thus expected to be applicable to regime for weak fields. To apply the analytical expressions to our simulation data, we use the rotational friction coefficient $\varGamma$ of the bacterium as a fit parameter. We know the friction coefficient of the cell body, but this might be modulated by the presence of the flagellum. Fitting the U-turn time with the expression from eq.(19), we find good agreement with the theoretical prediction for $\varGamma /(8\pi \eta R^3)=1.12$, i.e., for a friction coefficient of the whole bacterium that is increased compared to that of the cell body alone ($8\pi \eta R^3$).

To test this result, we also determined the friction coefficient directly but rotating the bacterium with an external torque. For this simulations, we turned off the motor torque and rotated the bacterium with an external torque applied to the cell body in a direction perpendicular to the motor axis. We then measured the angular velocity of the cell body to determine the friction coefficient. The inset in Fig. 5a shows the resulting estimate of the rotational friction coefficient of the whole bacterium as a function of its flagellum length. As expected, the drag coefficient is increased in the presence of a flagellum. The dependence on the flagellum length is relatively weak. The minimum in the friction coefficient coincides with the length where the flagellum has just one full pitch. The numerical value for a length of $L = 12 \,\upmu \hbox {m}$ is 1.19, close to what we have obtained from the fit to the U-turn time.

Figure 5d shows the diameter of the U-turn (scaled by the velocity) as a function of magnetic field for flagellum lengths of $L = 4, 6, 8 \,\upmu \hbox {m}$. The normalization by velocity is important because in populations of MTBs, the velocity can vary from cell to cell. Moreover, from an experimental point of view, the U-turn diameter and the velocity are more easily accessible than the U-turn time. According to Fig. 5d, the U-turn diameter also exhibits the two regimes discussed for the U-turn time. Fitting the data with Eq.(20) results in a friction coefficient of $\varGamma /(8\pi \eta R^3)=1.23$, slightly larger than the result from fitting the U-turn time, but in good agreement with the direct measurement of the friction coefficient. The discrepancy appears to be related to the fact that the theory assumes entirely planar motion, while in the simulations, the bacterium makes small excursions in the direction perpendicular to the main plane of motion during the U-turn.

### The effect of magnetic moment inclination on 3D motility pattern

In this section, we investigated how the swimming trajectory of our model bacterium is modified by an inclination of the magnetic moment with respect to the propulsion axis. To that end, we modulated the angle $\theta _m$ in our simulations (${0}^{\circ } \le \theta _{m} < {90^{\circ }}$). Small inclination angles are expected even for bacteria where the nominal direction of the magnetic moment is along the propulsion axis, as the precision of control is necessarily finite. However, some species including *Magnetococcus marinus* (MC-1) and *Magnetococcus massalia* (MO-1) do show rather large angles. These do, however, not have single flagellum as our model bacterium here and for MC-1, we proposed recently that it swims with a different body orientation that reduces this angle to some extent [9]. Nevertheless, it is interesting to quantitatively analyze the effect of such inclination.

**Fig. 6:**
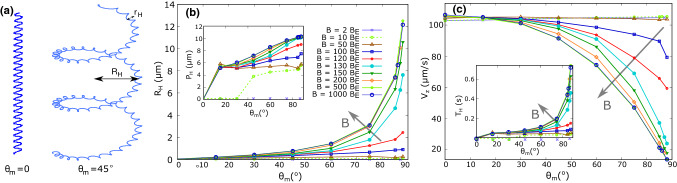
**a** Side view of the (double-)helical trajectories observed for the swimming of a model bacterium without and with inclined magnetic moment (at an angle $\theta _{m} = {45}^{\circ }$ relative to the flagellar axis and for $B= 500 B_E$). The direction of the magnetic field is upward. **b** Radius of the large helix, and **c** net swimming velocity in the direction of the magnetic field as functions of the angle between the magnetic moment and the flagellar propulsion axis ($\theta _{m}$) for different field strengths. The insets in **b** and **c** show the corresponding pitch and period of the large helix, respectively

When the magnetic moment is parallel to the flagellum axis ($\theta _{m} = {0^{\circ }}$), the case studied so far, the overall motion is observed to be approximately on a straight line. Closer inspection shows a wiggling of the cell body due to a weak rotational motion around that line, so that the trajectory is actually helical with a very small helix radius, smaller than the size of the cell body, as shown in Fig. 6a, where the radius is about $0.1\,\upmu \hbox {m}$. The incidence of such wiggling has been noted in earlier work and reflects the fact that the axis of propulsion is not exactly parallel to the axis of the cell body or, in our case, the magnetic moment [47–49]: Due to the noninteger number of turns in the helix of the flagellum, the thrust force always has a component normal to the axis of the flagellum and leads to an off-axis torque. Propulsion together with this off-axis torque result in wiggling. A comprehensive study that characterized the wiggling in microorganism trajectories was presented by Hyon et al. [49].

When the magnetic moment is not parallel to the flagellum ($\theta _{m} \ne {0}^{\circ }$), we observe double helical trajectories in the presence of a magnetic field. The small helix due to the wiggling is now bent and follows the path of a larger helix due to the precession of the magnetic moment with a radius in the order of several times that of the body size. An exemplary double helical trajectory is depicted in Fig. 6a for $\theta _{m} = {45}^{\circ }$ and $B= 500 B_E$. The radii of the small and large helices are represented with $r_H$ and $R_H$, respectively. The axis of the large helix is parallel to the direction of the magnetic field.

We characterized the double helical trajectories by the swimming velocity $V_z$ along the direction of the magnetic field (which we take to be in the *z* direction), and by the radius, pitch, and period of the large helix ($R_H$, $P_H$ and $T_H$, respectively). We extracted these parameters from the trajectories obtained for different inclination angles in the range of $\theta _m = 0-{88}^{\circ }$ and different strengths of the magnetic field ($B = 0 - 1000B_E$). The results for the helix radius ($R_H$) and the swimming velocity ($V_z$) are plotted in Fig. 6b, c as functions of the inclination angle $\theta _m$ for different strengths of the magnetic field. The corresponding plots for the pitch ($P_H$) and the period ($T_H$) are shown as insets in Fig. 6b, c.

Our results show that the swimming velocity decreases with increasing inclination angle $\theta _{m}$ (Fig. 6c), while the radius of the helix increases (Fig. 6b). Two regimes for low and high magnetic fields can be distinguished. At low magnetic fields ($< 50B_E$), the large helix is not easily detectable since it is only very slightly greater than the small helix (only if $\theta _m$ approach ${90^{\circ }}$). In this regime, the magnetic torque is not strong enough to cause considerable precession of the bacterium. As a consequence, the velocity is almost not affected by the inclination angle. For small inclinations ($\theta _{m}< {20}^{\circ }$), the swimming velocity is even slightly increased by the presence of the magnetic field, likely because the wiggling motion is slightly suppressed. We expect that this effect is more pronounced in the presence of thermal fluctuations, which provide stronger perturbations of the alignment with the field, which in turn restrains the bacterium to swim only in one dimension [16].

The large helix is clearly detectable for $B > 120B_E$ and $\theta _{m} > {45}^{\circ }$. In this case, the radius of large helix is about several times the body size and the velocity decreases strongly with the inclination angle. The strong magnetic torque forces the magnetic moment into a strong rotation toward the magnetic field and hence a greater deviation from the propulsion axis.

These observations indicate that small inclination angles ($<{30}^{\circ }$) due to imprecise orientation of the magnetic moments with respect to the propulsion axis do not affect the swimming of MTB in naturally occurring fields much. Even inclination angles of ${45}^{\circ }$ would not strongly hinder the motion. However, inclination angles exceeding ${45}^{\circ }$ as observed in strains MC-1 and MO-1 would put those cells at a disadvantage. This observation indicates that a different swimming mode with different orientation of the magnetic moments as proposed for MC-1 [9] is beneficial for these strains.

## Conclusion

In this work, we developed a detailed model for the propulsion and orientation of magnetotactic bacteria (MTB) based on Stokesian dynamics and investigated their swimming in the presence of an external magnetic field. There is a great variety of MTB in nature that differ in the structure of their motility apparatus (e.g., single- or multiflagellated with different localizations of the flagella on the cell surface) and the organization of their magnetosomes, the organelles providing them with a magnetic moment (single chain, multiple chains, different chain orientation) [15]. Here, we studied a model MTB with the simplest motility apparatus, a single flagellum, and varied its parameter as well as the orientation of the magnetic moment and the field strength (we note that a variation in the strength of the magnetic moment has the same effect as the corresponding change in the magnetic field strength, as only their product enters the orientational dynamics). In the simulation, we have the possibility to investigate a wide range of values for these parameters, some of which are not biologically relevant but worth studying as they might be applicable for predicting swimming behavior in the case of artificial microsiwmmers.

After an initial characterization of our minimal model bacterium, we considered two scenarios that are of interest for the experimental study of magnetotactic bacteria. On the one hand, we simulated U-turns upon reversal of the magnetic field direction, a scenario often used to measure the magnetic moment of the bacteria [44]. On the other hand, we studied the three-dimensional shape of swimming trajectories in a magnetic field, a quantity that has become accessible with the introduction of 3d tracking methods [50] and that provides information about swimming mechanisms as shown recently for the *Magnetococcus marinus* MC-1 [9].

With respect to U-turns, our simulations qualitatively agree with a theoretical description based on a moving point dipole (at least for the weak fields relevant to experiments). However, they also point toward a difficulty of using U-turns to determine the magnetic moment of a bacterium, namely that the rotational friction coefficient must be known. Typically, a friction coefficient is estimated for the geometry of the cell body, but this may be a cause of inaccuracy as indicated by our simulations. For strong fields, the U-turns in our simulation do not follow the prediction of the point dipole theory, because the relaxation of the flagellum (which determined the direction of propulsion) is slower than the relaxation of the magnetic moment. Thus, our results suggest that using stronger fields than typically used in experiments may result in novel behaviors such as formation of kink in flagellum during a U-turn as well as the observation of two different characteristic U-turn times. These should provide insights into the elastic response of the flagellum. However, we also note that with these strong deformations of the flagellum, other flagellar properties may come into play, in particular polymorphic transitions [20, 34, 51].

With respect to swimming trajectories, we observed bacteria with a magnetic moment at a non-zero angle relative to the flagellar axis to swim on double helical trajectories, spiralling in the direction defined by the magnetic field. Helical trajectories are seen in various microorganisms and are of interest for different swimming and sensing strategies [9, 18, 30, 52–54]. When we characterized the swimming velocity on these trajectories, we found that small inclination angles ($\theta _{m}< {20}^{\circ }$) are not a significant disadvantage for swimming in realistic field strength. Large inclinations, as seen in some MTB such as strain MC-1, would be a problem, as the net velocity in the direction of the field is considerably reduced by the helical motion. However, our recent study [9] suggests that strain MC-1 avoids this problem by using a different swimming mechanism (cooperative pushing and pulling flagella), which reduces the inclination of the magnetic moment with respect to the propulsion axis.

The simulation method presented here is quite general and has indeed been used for different types of swimmers, specifically *Escherichia coli* with multiple flagella all over the cell body [33], *Shewanella putrefaciens* with a single flagellum [8] and *Magnetococcus marinus* with two flagella and a magnetic moment [9]. Since the model includes both the elasticity of the flagellum and hydrodynamic interactions, it is particularly suited to address questions of re-orientation as well as flagellar synchronization in multi-flagellated microorganisms [33, 55, 56].

Our model can also be used to characterize the swimming of MTB for their potential applications in MTB-inspired microrobots. For example, having a precise control on the helical trajectory might be useful in enhancing the bacterium sensing of external stimuli [49]. One can also extend the model to include a cargo to be transported by the bacterium and study its effect on the swimming bacterium. In general, our results here and those in our previous work [9] show that Stokesian dynamics provides a useful method to address the diverse swimming mechanisms of magnetotactic bacteria.

## Supplementary Information

Below is the link to the electronic supplementary material.

Supplementary material 1 (mpg 2638 KB)
